# High Summer Temperatures and Mortality in Estonia

**DOI:** 10.1371/journal.pone.0155045

**Published:** 2016-05-11

**Authors:** Daniel Oudin Åström, Christofer Åström, Kaidi Rekker, Ene Indermitte, Hans Orru

**Affiliations:** 1 Department of Family Medicine and Public Health, University of Tartu, Tartu, Estonia; 2 Department of Public Health and Clinical Medicine, Occupational and Environmental Medicine, Umeå University, Umeå, Sweden; 3 Centre for Primary Health Care Research, Department of Clinical Science, Malmö, Lund University, Lund, Sweden; 4 Tartu Health Care College, Tartu, Estonia; Columbia University, UNITED STATES

## Abstract

**Background:**

On-going climate change is predicted to result in a growing number of extreme weather events—such as heat waves—throughout Europe. The effect of high temperatures and heat waves are already having an important impact on public health in terms of increased mortality, but studies from an Estonian setting are almost entirely missing. We investigated mortality in relation to high summer temperatures and the time course of mortality in a coastal and inland region of Estonia.

**Methods:**

We collected daily mortality data and daily maximum temperature for a coastal and an inland region of Estonia. We applied a distributed lag non-linear model to investigate heat related mortality and the time course of mortality in Estonia.

**Results:**

We found an immediate increase in mortality associated with temperatures exceeding the 75^th^ percentile of summer maximum temperatures, corresponding to approximately 23°C. This increase lasted for a couple of days in both regions. The total effect of elevated temperatures was not lessened by significant mortality displacement.

**Discussion:**

We observed significantly increased mortality in Estonia, both on a country level as well as for a coastal region and an inland region with a more continental climate. Heat related mortality was higher in the inland region as compared to the coastal region, however, no statistically significant differences were observed. The lower risks in coastal areas could be due to lower maximum temperatures and cooling effects of the sea, but also better socioeconomic condition. Our results suggest that region specific estimates of the impacts of temperature extremes on mortality are needed.

## Introduction

On-going climate change is predicted to result in a growing number of extreme meteorological events—such as heat waves—throughout Europe [1]. The effect of high temperatures and heat waves are already having an important impact on public health in terms of increased mortality and morbidity [2–5], with heightened susceptibility reported among the elderly [6] and groups with chronic disease [7]. Increasing mortality and morbidity due to elevated temperatures has been reported in Southern and Western Europe [2, 3, 5], Central Europe [8], and in the colder climates of Northern Europe such as in nearby Sweden and Finland [5, 7, 9].

During the 2010 heat wave in Eastern Europe and Russia 2010 unprecedented temperatures were recorded [10]. The impacts upon public health in those countries—including Estonia—due to the 2010 heat waves were substantial [11]. The adverse effects of heat on health are usually a direct effect with increased mortality on the day of or one or two days after high temperatures [4, 12]. Elevated mortality rates persist throughout the length of a heat wave [9]. A reduction in mortality following a heat wave has been observed in some studies, a phenomenon called mortality displacement or harvesting [13–15]. The increased mortality during the 2010 heat wave in Russia was followed by months of lower than normal mortality in Moscow [16].

Increased health risks in relation to high temperatures tends to be greater in colder climates, whereas to a larger extent cold temperatures tend to increase health risks in warmer climates [12, 17]. This may suggest a partial adaptation of populations to their local climate [12, 17]. The effect of temperature on mortality can vary substantially, both within a country and populations. In Italy, region of birth has been associated with heat sensitivity in adulthood [18]. Baccini et al. (2008) reported substantial heterogeneity for the effect of high temperatures on mortality among 15 European cities with very different climates [17]. Michelozzi et al. (2009) reported heterogeneity for the relationship between temperature and morbidity [2]. At country level, some heterogeneity of the effect of high temperatures on mortality has been reported between regions in England and Wales [19] and Italy [20, 21].

The current study aimed to investigate heat related mortality and the time course of such mortality, for a coastal and an inland region of Estonia, as well as on a country level. Furthermore, we explored whether age and gender modified the relationship between heat and mortality in Estonia.

## Materials and Methods

**Fig 1:**
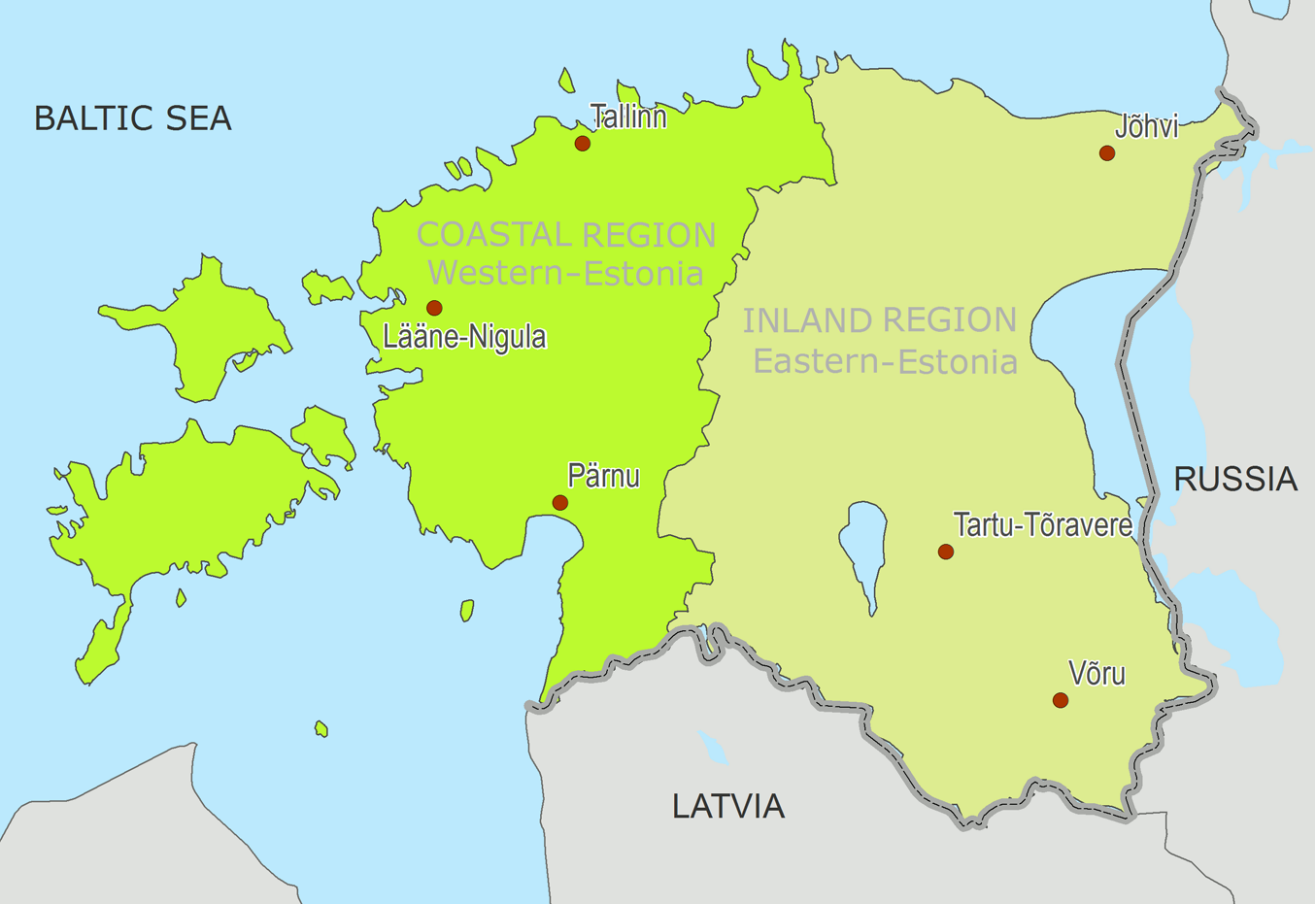
The coastal and inland regions of Estonia, with the meteorological stations used in the current study.

Estonia is situated in north-eastern Europe bordered by the Baltic Sea Finland, Latvia and the Russian Federation. Estonia is in the northern part of the temperate climate zone and a transition zone between maritime and continental climates. In the current research, Estonia was divided into a coastal region (Western-Estonia with a more coastal climate) and an inland region (Eastern-Estonia with a more continental climate) (Fig 1).

Daily temperature data were acquired from the Estonian Weather Service for seven meteorological stations—three in each region and one for centrally located assuming exposure for Estonia as a whole (Türi)—for the four warmest summer months (June until September) for the period 1997–2013 (Fig 1). We used maximum daily temperature as our exposure variable. Daily maximum temperature per region was calculated as the average (mean) of the maximum temperatures from the three stations. Temperature data was complete for all meteorological stations but one. On the 7^th^ and 8^th^ of June 2012 observations were missing for the measuring station Lääne-Nigule, on those two occasions regional average maximum temperatures were calculated as the average of the two remaining stations.

Daily all-cause mortality was calculated for the same period for the entire country. Data on deaths was acquired from the Estonian Causes of Death Registry, which is a national digitized database overseen by the National Institute for Health Development. For every death the following data was obtained: place of residence; date of death; gender; age. Total population data as of the 1^st^ of January for each studied year was retrieved from Statistics Estonia. Based on the number of deaths and population data, we calculated yearly mortality rates for both regions and Estonia.

To investigate any potential effect modification by age and gender, the analyses were stratified accordingly. The following age groups were investigated: 0–74 and 75+.

### Statistical methods

Temperature-mortality relationships for the summer months during the period 1997–2013 were derived using a generalised linear model (allowing for overdispersion) for each region. Given the non-linear as well as delayed relationship between temperature and mortality this relationship was modelled using a distributed lag non-linear model [22, 23]

$$Y_{t}∼Poisson\left(\mu_{t}\right)$$

$$\text{log}\left(\mu_{t}\right)\;=\;\alpha \;+\;{\beta T}_{t,l}\;+\;{weekday}_{t}\;+\;{holiday}_{t}\;+NS\left({doy}_{t},\;df\;=\;3\;per\;year\;\right)+NS\left(trend,\;df\;=\;1\;per\;year\right)$$

where Y_t_ was the number of deaths a day per region, α the intercept and βT_t,l_ the vector of coefficients that represented the non-linear and delayed relationship between daily maximum temperature and daily mortality. A quadratic B-spline and a natural cubic spline was fitted for temperature and time lag (up to 10 days) respectively. For temperature we used two equally spaced internal knots and for the time lag two equally spaced knots on the log-scale. Weekday_t_ was a categorical variable for the specific day of the week and holiday_t_ a binary variable indicating Estonian public holidays. Natural cubic splines with three and one degree of freedom per year allowed to take into account variability in mortality owing to seasonality and longer term time trends respectively (chosen according to the lowest Akaike Information Criteria).

As sensitivity analyses we extended the lag period up to 14 and 21 days.

The minimum mortality temperature (MMT) occurred at approximately the 75^th^ percentile (≈ 24°C) of the daily maximum temperature distribution during the summer months for Estonia (Türi station) and the inland region. The 75^th^ percentile of the regional temperature distribution was used as a centring point for the analyses (12).

To generate an estimate for Estonia as a whole we used two approaches. First we pooled the regions-specific estimates using fixed effects meta analyses [24]. Secondly, we assumed one centrally located meteorological station, Türi, to represent exposure for Estonia and derived the estimates through time series regression as described above.

R version 2.13.1 was used to create datasets of the variables and the DLNM and MVMETA add-on packages were used for statistical modelling. The data and R-code used are available online (S1 and S2 Files).

## Results

The meteorological data showed similar temperatures between the coastal and inland regions, with the inland region experiencing higher and lower extreme values (Table 1). Meteorological data per measuring station is available in S1 Table.

**Table 1:** Daily maximum temperatures for the summer months over the period 1997–2013 per region.

	MEAN	SD	MIN	MAX	75^th^ percentile	90^th^ percentile	99^th^ percentile
ESTONIA^a^	20.8	4.6	5.6	33.4	23.8	27.0	30.4
COASTAL	20.3	4.2	7.9	32.4	23.0	26.0	30.2
INLAND	20.5	4.6	6.0	34.3	23.6	26.6	31.2

a) Estonia measurements are based on one centrally located meteorological station, Türi.

The number of deaths that occurred during the study period were evenly distributed between: the two age categories; the genders; each region (Table 2). In total there were 91,673 deaths during the studied period.

**Table 2:** Average mortality data for the period 1997–2013.

	Size of population	Annual mortality rate	Daily number of deaths					Distribution of deaths	
	N Total	Per 1000 inhabitants	N Total	MEAN	MEDIAN	MIN	MAX	% 75+	% Female
ESTONIA	1,360,719	12.7	91,673	44.2	44	21	73	45.7	48.5
COASTAL	741,237	11.5	45,429	22.1	22	8	43	45.9	50.4
INLAND	619,482	14.0	15,682	22.0	22	8	48	45.5	49.6

Mortality significantly increased owing to high summer temperatures. The cumulative relative risks (RR) over lags 0–2 for the 75^th^ vs. the 99^th^ percentiles of daily maximum temperature, was 1.12 (95% Confidence Interval (CI): 1.05–1.21) in the coastal region and 1.28 (95% CI: 1.20–1.37) in the inland region. The estimated RRs for the different age groups (0–74, 75+) were similar in the coastal region. In the inland region 75+ year olds had a significantly higher RR estimate than those <75 years. Gender was not found to modify the risk of mortality in either region. The cumulative RRs over lags 0–10 shows similar patterns. (Table 3). The RRs estimated by meta-analyses also show increased short term mortality due to high temperatures and are similar to the estimates generated assuming Türi station to represent temperature exposure for entire Estonia, although with wider CIs, thus statistically increased mortality were not found for all of the investigated groups.

**Table 3:** Cumulative Relative Risks over lags 0–2 and lags 0–10 with 95% Confidence Intervals for the 75^th^ vs. 99^th^ percentiles per region.

Lags 0–2	Total	Male	Female	0–74 years	75+ years
Estonia Turi	1.18 (1.13–1.24)	1.17 (1.09–1.24)	1.20 (1.13–1.28)	1.14 (1.08–1.22)	1.15 (1.10–1.21)
Estonia Meta	1.18 (1.03–1.34)	1.18 (1.08–1.29)	1.18 (0.98–1.42)	1.13 (1.07–1.20)	1.24 (0.96–1.59)
Coastal	1.12 (1.05–1.21)	1.16 (1.05–1.27)	1.10 (0.99–1.21)	1.13 (1.03–1.25)	1.11 (1.01–1.23)
Inland	1.28 (1.20–1.37)	1.24 (1.13–1.36)	1.33 (1.20–1.46)	1.15 (1.04–1.26)	1.45 (1.31–1.60)
Lags 0–10	Total	Male	Female	0–74 years	75+ years
Estonia Turi	1.13 (1.06–1.20)	1.15 (1.05–1.27)	1.10 (1.00–1.22)	1.13 (1.04–1.23)	1.12 (1.03–1.23)
Estonia Meta	1.12 (0.97–1.29)	1.17 (1.08–1.27)	1.06 (0.80–1.41)	1.13 (1.04–1.22)	1.11 (0.79–1.56)
Coastal	1.06 (0.97–1.17)	1.20 (1.05–1.38)	0.92 (0.80–1.06)	1.18 (1.03–1.34)	0.93 (0.81–1.07)
Inland	1.25 (1.13–1.38)	1.22 (1.06–1.40)	1.29 (1.11–1.49)	1.15 (1.00–1.32)	1.38 (1.19–1.60)

**Fig 2:**
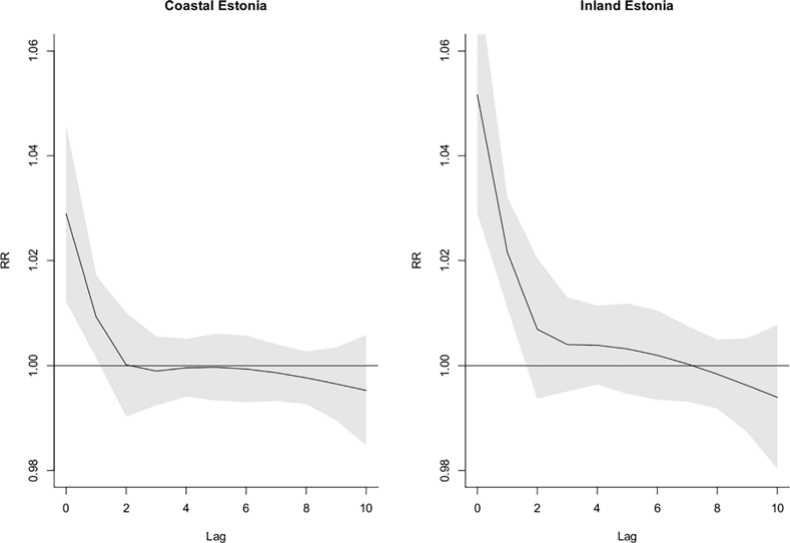
The estimated RR of total mortality at the 90^th^ percentile of daily maximum temperature for lags 0–10 for the coastal and inland region of Estonia.

Temperature had an immediate effect on mortality. We found increased risks of mortality in both regions the same day and the day after exposure to elevated temperatures. No strong evidence of mortality displacement was found in either region (Fig 2). When extending the lag period up to 21 days there was after 17 days some evidence suggesting lower than normal mortality lasting for four days in the coastal region, however the difference between the regions was not statistically significant (results not shown).

For the coastal region, MMT was found at approximately the 10^th^ percentile (15°C) of the coastal region temperature distribution. In the inland region MMT was found at approximately the 75^th^ percentile (24°C) of the inland region temperature distribution. The cumulative RR for MMT vs. the 99^th^ percentile in the coastal region was 1.20 (1.10–1.30) for lags 0–2 and 1.10 (0.98–1.23) for lags 0–10.

**Fig 3:**
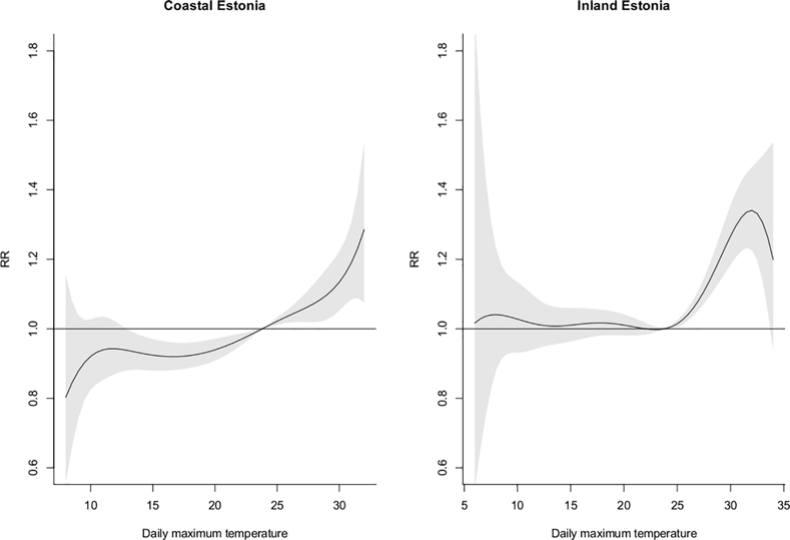
The estimated cumulative RR of total mortality for the daily max temperature over lags 0–2 for the coastal and inland region.

The cumulative RRs over lags 0–2 reveals increased mortality due to high summer temperatures in both regions and the considerably lower MMT in the coastal region (Fig 3).

**Fig 4:**
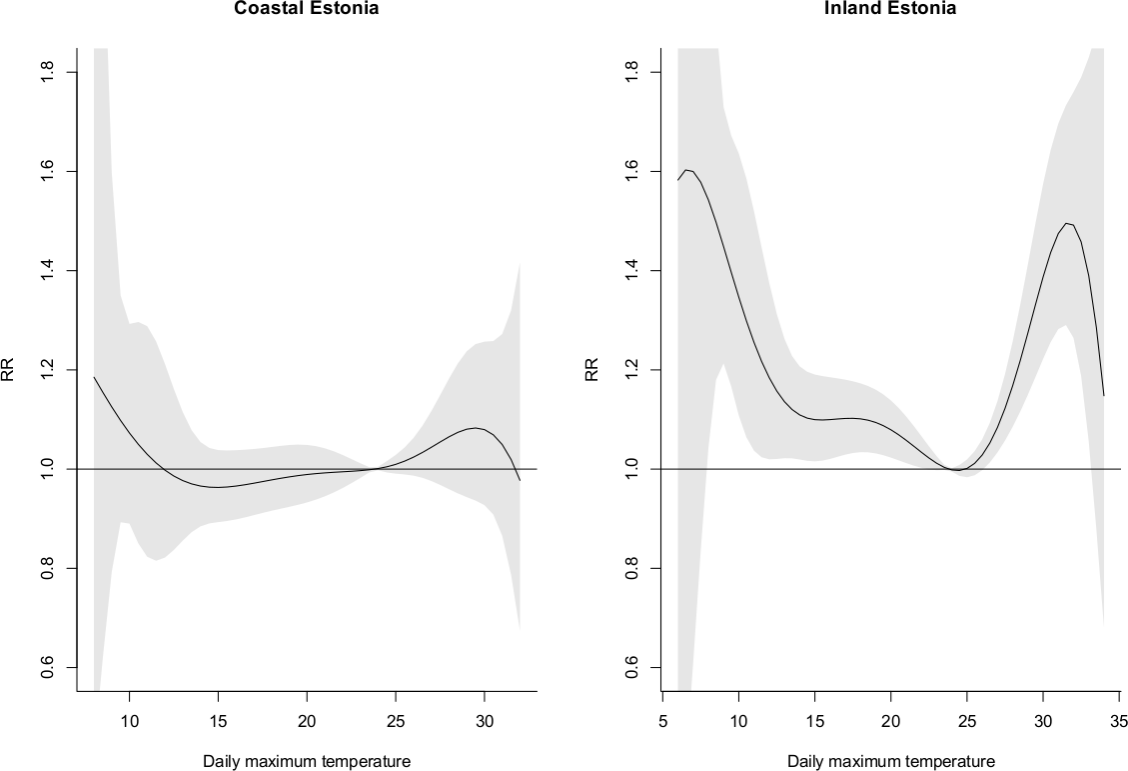
The estimated cumulative RR of total mortality for the daily max temperature over lags 0–10 for the coastal and inland region.

When extending the lag period up to ten days, the cumulative RRs indicated non-significant increase in total mortality increased mortality across the temperature distribution. Total mortality increased due to high and modestly cold summer temperatures in the inland region. The differences between regions were not statistically significant (Fig 4).

## Discussion

We found increased mortality for temperatures exceeding the 75^th^ percentile of the region specific temperature distribution; this increase lasted for 1–2 days and occurred in both regions. The immediate effects of high temperatures were of a larger magnitude in the inland region compared to the coastal region, although the cumulative RRs were not statistically significantly higher. As mean daily maximum summer temperatures were similar in both regions, the coastal climate might be easier on susceptible people. The short term effects of temperature on mortality as manifested by the RRs presented for lags 0–2 in the present study are similar to what has been reported for nearby Sweden and Finland [5].

The RRs estimated by meta-analyses also show statistically significantly increased short term mortality due to high temperatures for total mortality as well as male and 75 plus mortality. The point estimates derived by meta-analyses were similar to the estimates generated assuming Türi station to represent temperature exposure for entire Estonia. Using the latter approach, we found statistically significantly increased mortality for all groups. However, pooling the estimates does not reveal differences between the regions, for instance the lower MMT in the coastal area, suggesting that regional specific estimates may improve actions targeted at reducing the impact of heat and heat waves on public health.

MMTs were found at approximately the 75^th^ percentile of the temperature distribution for both Estonia as a whole and the inland region. However, the MMT in the coastal region was considerably lower and found at approximately the 10^th^ percentile. Thus we will be underestimating the effect of heat on mortality in the coastal region when only considering the 75^th^ vs. the 99^th^ percentiles, as more modest summer temperatures will also contribute to heat related mortality. The RRs derived when comparing MMT vs. the 99^th^ percentile in the coastal region were more similar to the RRs for the inland region.

Our finding of a MMT at such a high percentile of the temperature distribution for Estonia and the inland region is in line with previous studies for similar climates [25, 26]. MMT in the coastal region was found at approximately the 10^th^ percentile and this is in line with what have previously been reported on a country level in Spain, with a warmer climate [27]. These differences may be due to the modelling choice. Our estimated MMTs would likely change if the temperature-mortality relationship was modelled differently or the degrees of freedom used in the modelling stage were changed.

Our findings that the inland risks may be of a larger magnitude are consistent with a recent analysis of temperature vulnerability of the population in Estonia, where higher vulnerability on hot days (daily maximum above 27°C) was reported for inland inhabitants as compared to those of coastal regions [28]. Roose et al (2015) reported that the main drivers of higher vulnerability were larger proportions and density of children (under four years old) and the elderly (above 65 years), poor socioeconomic status, and a larger proportion of built-up areas [28].

Effect modification by age and gender was not present in our data, as the CIs were overlapping. In the coastal region women had a higher RR than men and the oldest age group (75+) had a lower RR the age group 0–75, whereas in the inland region this was the opposite. Even though the differences were not statistically significantly different, such analyses may improve regional knowledge regarding groups with a heightened susceptibility to heat exposure.

The total effect of elevated temperatures on mortality was not lessened by significant mortality displacement in either region or Estonia as a whole. When extending the lag period up to 21 days there was after 17 days some evidence suggesting lower than normal mortality lasting for four days in the coastal region, however the difference between the regions was not statistically significant. Significant mortality displacement has been reported in a few studies conducted in Eastern Europe. Kyselý (2004) reported mortality displacement following heat waves in the Czech Republic, [15] and Shaposhnikov et al. (2015) reported that mortality displacement in Moscow occurred over the months following the 2010 heat wave [16].

Even though our finding of suggestive differences in heat related mortality between regions, e.g. considerably lower MMT in the coastal region and generally higher risk estimates in the inland region, is in line with e.g. studies that showed heat related mortality differed between regions in Italy [20, 21], our results should be interpreted with caution. There might even be intra-regional differences, e.g. the coastal region includes Tallinn, the capital of Estonia, where more than half of the region’s population lives. Hondula et al. (2013) have shown that even within a city different mortality rates can occur, which suggests that identification of high-risk areas is important from a public health perspective [29].

Limitations: The temperature data collected from the different meteorological stations were assumed to represent exposure, but there is scope for exposure misclassification on an individual level when one considers the entirety of each region [30]. For example, assigning the same exposure level throughout an entire region based on a few measurement locations could lead to an over- or under-estimation of the risk of dying during episodes of high temperatures. Furthermore, as the meteorological stations were situated outside of cities, the actual temperature exposures in cities may have be underestimated owing to the urban heat island effect. Also, indoor and outdoor temperatures may be poorly correlated due to a number of modifying factors such as e.g. air conditioning [31]. Time indoors will alter individuals’ exposure, and there are a number of other factors that might have a similar impact on personal exposure levels. Workplace conditions and type of transportation might have an even larger impact on personal exposure level. The choice of temperature metric used in our study—maximum daily temperature—should reflect exposure at a population level and not have significantly influenced our results. Correlations between different temperature metrics, such as the daily minimum, mean and maximum temperature were strong and they should, on average, be equally well suited for studies similar to the present one [32].

## Conclusions

We observed statistically significantly increased mortality following a period of high temperature for an Estonian coastal region and an inland region, as well as on a country level. Even though the differences between regions were not statistically significant the considerably lower MMT in the coastal region and the generally higher RRs in the inland region suggests regional estimates of the temperature-mortality relationship may be of public health interest. Effect modification by age or gender was not present in our data.

## Supporting Information

S1 File Data contains the following variables: Date variables: Date, Year, Month, Day, doy (Day of Year, variable taking on the value 1 the 1st of June each year and the value 122 the 30th of September each year), Wday (categorical variable indicating day of week), Hday (binary variable indicating public Estonian holiday or not), Trend (trend variable used in the smooth function for trend). Exposure variables: Maxtemp_i (Daily maximum temperatures for Estonia and each region i = 1–3 (i = 1 Estonia (Türi Station), 2 = Coastal region, 3 = Inland region)Mortality variables: N_ij, (the daily number of deaths in each investigated region and group, i = 1–3 (i = 1 Estonia (Türi Station), 2 = Coastal region, 3 = Inland region) j = 1–5 (i = 1 Total Mortality, 2 = Male Mortality, 3 = Female Mortality, 4 = <75 Mortality, 5 = 75+ Mortality)).
(TXT)

S2 File R-code. (DOCX)

S1 Table Daily maximum temperatures for the summer months over the period 1997–2013 per meteorological station. (DOCX)

## References

[1] Field CB. Managing the risks of extreme events and disasters to advance climate change adaptation: special report of the intergovernmental panel on climate change: Cambridge University Press; 2012. 10.1136/jech-2012-201045 22766781

[2] Michelozzi P, Accetta G, De Sario M, D'Ippoliti D, Marino C, Baccini M, et al High temperature and hospitalizations for cardiovascular and respiratory causes in 12 European cities. American journal of respiratory and critical care medicine. 2009;179(5):383–9. 10.1164/rccm.200802-217OC 19060232

[3] D'Ippoliti D, Michelozzi P, Marino C, de'Donato F, Menne B, Katsouyanni K, et al. Research The impact of heat waves on mortality in 9 European cities: results from the EuroHEAT project. 2010. 10.1186/1476-069X-9-37 PMC2914717 20637065

[4] Guo Y, Gasparrini A, Armstrong B, Li S, Tawatsupa B, Tobias A, et al Global variation in the effects of ambient temperature on mortality: a systematic evaluation. Epidemiology. 2014;25(6):781–9. 10.1097/EDE.0000000000000165 25166878 PMC4180721

[5] de’Donato FK, Leone M, Scortichini M, De Sario M, Katsouyanni K, Lanki T, et al Changes in the effect of heat on mortality in the last 20 years in nine European cities. Results from the PHASE project. International journal of environmental research and public health. 2015;12(12):15567–83. 10.3390/ijerph121215006 26670239 PMC4690942

[6] Oudin Åström D, Bertil F, Joacim R. Heat wave impact on morbidity and mortality in the elderly population: a review of recent studies. Maturitas. 2011;69(2):99–105. 10.1016/j.maturitas.2011.03.008 21477954

[7] Åström DO, Schifano P, Asta F, Lallo A, Michelozzi P, Rocklöv J, et al The effect of heat waves on mortality in susceptible groups: a cohort study of a mediterranean and a northern European City. Environmental Health. 2015;14(1):30. 25889290 10.1186/s12940-015-0012-0 PMC4397690

[8] Hanzlíková H, Plavcová E, Kynčl J, Kříž B, Kyselý J. Contrasting patterns of hot spell effects on morbidity and mortality for cardiovascular diseases in the Czech Republic, 1994–2009. International journal of biometeorology. 2015:1–12. 10.1007/s00484-015-0974-1 25744153

[9] Rocklov J, Ebi K, Forsberg B. Mortality related to temperature and persistent extreme temperatures: a study of cause-specific and age-stratified mortality. Occup Environ Med. 2011;68(7):531–6. 10.1136/oem.2010.058818 . 20962034

[10] Barriopedro D, Fischer EM, Luterbacher J, Trigo RM, García-Herrera R. The hot summer of 2010: redrawing the temperature record map of Europe. Science. 2011;332(6026):220–4. 10.1126/science.1201224 21415316

[11] Rekker K Indermitte E, Saava A. The extraordinarily hot summer of 2010 in Estonia and its impact on all-cause mortality. Eesti Arst. 2013;92(2)(40):(In Estonian).

[12] Anderson BG, Bell ML. Weather-related mortality: how heat, cold, and heat waves affect mortality in the United States. Epidemiology (Cambridge, Mass). 2009;20(2):205. 10.1097/EDE.0b013e318190ee08 PMC3366558 19194300

[13] Hajat S, Armstrong BG, Gouveia N, Wilkinson P. Mortality displacement of heat-related deaths: a comparison of Delhi, Sao Paulo, and London. Epidemiology. 2005;16(5):613–20. 16135936 10.1097/01.ede.0000164559.41092.2a

[14] Saha MV, Davis RE, Hondula DM. Mortality Displacement as a Function of Heat Event Strength in 7 US Cities. American journal of epidemiology. 2014;179(4):467–74. 10.1093/aje/kwt264 24264293

[15] Kyselý J. Mortality and displaced mortality during heat waves in the Czech Republic. International Journal of Biometeorology. 2004;49(2):91–7. 15549422 10.1007/s00484-004-0218-2

[16] Shaposhnikov D, Revich B, Bellander T, Bedada GB, Bottai M, Kharkova T, et al Long-Term Impact of Moscow Heat Wave and Wildfires on Mortality. Epidemiology. 2015;26(2):e21–e2. 10.1097/EDE.0000000000000251 25643114

[17] Baccini M, Biggeri A, Accetta G, Kosatsky T, Katsouyanni K, Analitis A, et al Heat effects on mortality in 15 European cities. Epidemiology. 2008;19(5):711–9. 10.1097/EDE.0b013e318176bfcd 18520615

[18] Vigotti MA, Muggeo VM, Cusimano R. The effect of birthplace on heat tolerance and mortality in Milan, Italy, 1980–1989. International journal of biometeorology. 2006;50(6):335–41. 16807711 10.1007/s00484-006-0035-x

[19] Hajat S, Kovats RS, Lachowycz K. Heat-related and cold-related deaths in England and Wales: who is at risk? Occupational and Environmental Medicine. 2007;64(2):93–100. 16990293 10.1136/oem.2006.029017 PMC2078436

[20] Michelozzi P, De Sario M, Accetta G, de’Donato F, Kirchmayer U, D’Ovidio M, et al Temperature and summer mortality: geographical and temporal variations in four Italian cities. Journal of epidemiology and community health. 2006;60(5):417–23. 16614332 10.1136/jech.2005.040857 PMC2563963

[21] Schifano P, Leone M, De Sario M, de’Donato F, Bargagli AM, D’Ippoliti D, et al Changes in the effects of heat on mortality among the elderly from 1998–2010: results from a multicenter time series study in Italy. Environ Health. 2012;11(1):58. 22943217 10.1186/1476-069X-11-58 PMC3506566

[22] Gasparrini A, Armstrong B, Kenward M. Distributed lag non‐linear models. Statistics in medicine. 2010;29(21):2224–34. 10.1002/sim.3940 20812303 PMC2998707

[23] Gasparrini A. Modeling exposure–lag–response associations with distributed lag non‐linear models. Statistics in Medicine. 2014;33(5):881–99. 10.1002/sim.5963 24027094 PMC4098103

[24] Gasparrini A, Armstrong B. Reducing and meta-analysing estimates from distributed lag non-linear models. BMC Medical Research Methodology. 2013;13(1):1. 23297754 10.1186/1471-2288-13-1 PMC3599933

[25] Gasparrini A, Guo Y, Hashizume M, Lavigne E, Zanobetti A, Schwartz J, et al Mortality risk attributable to high and low ambient temperature: a multicountry observational study. The Lancet. 2015. 10.1016/S0140-6736(14)62114-0 PMC4521077 26003380

[26] Åström DO, Tornevi A, Ebi KL, Rocklöv J, Forsberg B. Evolution of Minimum Mortality Temperature in Stockholm, Sweden, 1901–2009. 10.1289/ehp.1509692 26566270 PMC4892916

[27] Gasparrini A, Guo Y, Hashizume M, Kinney PL, Petkova EP, Lavigne E, et al Temporal variation in heat–mortality associations: a multicountry study. 2015 10.1289/ehp.1409070 25933359 PMC4629745

[28] Roose A SM, Kamenjuk P, Rosenthau A, Orru H, Tammepuu A. Assessment of climate change impacts and elaboration adaptation instruments in the field of planning, land use, health and rescue management: final report University of Tartu 2015.

[29] Hondula DM, Davis RE, Rocklöv J, Saha MV. A time series approach for evaluating intra-city heat-related mortality. Journal of epidemiology and community health. 2013;67(8):707–12. 10.1136/jech-2012-202157 23618771

[30] Armstrong BG. Effect of measurement error on epidemiological studies of environmental and occupational exposures. Occupational and Environmental Medicine. 1998;55(10):651–6. 9930084 10.1136/oem.55.10.651 PMC1757516

[31] Franck U, Kruger M, Schwarz N, Grossmann K, Roder S, Schlink U. Heat stress in urban areas: Indoor and outdoor temperatures in different urban structure types and subjectively reported well-being during a heat wave in the city of Leipzig. Meteorologische Zeitschrift. 2013;22(2):167–77.

[32] Barnett AG, Tong S, Clements A. What measure of temperature is the best predictor of mortality? Environmental research. 2010;110(6):604–11. 10.1016/j.envres.2010.05.006 20519131

