# Supplementary material for: High Summer Temperatures and Mortality in Estonia
Source: PLoS One. 2016 May 11;11(5):e0155045. doi: 10.1371/journal.pone.0155045 (PMC4864204; doi:10.1371/journal.pone.0155045)
Supplement: S2 File — (DOCX) [file pone.0155045.s002.docx]

Supplementary Material 1. R-code

## Define input data for Crossbasis

cen <- quantile(analys$maxtemp1,c(0.75))
lag = 10
varknots <- equalknots(analys$temp1,fun="bs",df=4,degree=2)
lagknots <- logknots(10,2)
argvar <- list(fun="bs",degree=4,knots=varknots,cen=cen)
arglag <- list(knots=lagknots)
bound <- range(analys$temp1)

Where maxtemp1 is the measurements assuming exposure for Estonia (Türi Station),

#Create the crossbases

cbi <- crossbasis(analys$maxtemp_i, lag=lag,
 argvar=argvar,
 arglag=arglag,
 group = analys$year)

i =1-3 (i=1 Estonia (Türi Station), 2=coastal region, 3=inland region)

#Run the models

Modij <- glm(N_ij ~ cbi + as.factor(wday) + hday +
 ns(trend, df=length(table(analys$year))*1) +
 ns(doy,df=length(table(analys$year))*3),
 family=quasipoisson(link=log), data = analys)

where N_ij is the daily number of deaths in each investigated region and group,
i=1-3 (i=1 Estonia (Türi Station), 2=Coastal region, 3=Inland region)
j=1-5 (i=1 Total Mortality, 2= Male Mortality, 3=Female Mortality, 4= <75 Mortality, 5= 75+ Mortality)
wday is a categorical variable for day of week,
hday is a binary variable indicating public Estonian holiday,
doy is a count variable for the day of year taking on the value 1 the first of May and 122 the 30^th^ of September each year.

# The predtemps

predij.temp <- crosspred(cbi, modij, cumul=TRUE, by=0.1)

i=1-3 (i=1 Estonia (Türi Station), 2=Coastal region, 3=Inland region)
j=1-5 (i=1 Total Mortality, 2= Male Mortality, 3=Female Mortality, 4= <75 Mortality, 5= 75+ Mortality)

#Find the cumulative RRs for lag02 (similar for lag010)

#Create lag02 variable for the temperature variable of interest.

xi <- apply(Lag(analys$tempi, c(0:2)), 1 , mean)

i=1-3 (i=1 Estonia (Türi Station), 2=coastal region, 3=inland region)

# Identify the 75th and 99th percentiles for lag02

q.e <- round(quantile(xi, probs = c(0.75, 0.99), na.rm = T), digits= 1)

# Extract the estimates and calculate the cumulative relative risks for lag20

RRij <- predij.temp$cumRRfit[which(as.numeric(rownames(predij.temp$cumRRfit))%in%q.e),]
RRijse <- predij.temp$cumse[which(as.numeric(rownames(predij.temp$cumse))%in%q.e),]

RelRij <- c((RRij[2,3])/(RRij[1,3]),
 exp( ( log(RRij[2,3])- log(RRij[1,3]) - (qnorm(.975)*(sqrt( (RRijse[2,3])^2+(RRijse[1,3])^2) ) ) ) )
 exp( ( log(RRij[2,3])- log(RRij[1,3]) + (qnorm(.975)*(sqrt( (RRijse[2,3])^2+(RRijse[1,3])^2) ) ) ) ))

i=1-3 (i=1 Estonia (Türi Station), 2=Coastal region, 3=Inland region)
j=1-5 (i=1 Total Mortality, 2= Male Mortality, 3=Female Mortality, 4= <75 Mortality, 5= 75+ Mortality)

#META ANALYSIS (example for lag02 and total mortality)

# Input meta analysis
q.m <- round(quantile(c(x2,x3), probs = c(0.75, 0.99), na.rm = T), digits= 1)

# Crossbasis input data (same as defined above)

# Meta lag 02

{yall <- matrix(NA,2,6)
Sall <- vector("list",2)
qaic <- 0
ylim = c(0,3)

# Coastal

{ # Cross bases and first stage models defined above
# REDUCTION TO SUMMARY ASSOCIATIONS, TO OVERALL CUMULATIVE SUMMARY
crall <- crossreduce(cb2,Mod_21_, lag = c(0,2))

# STORE THE RESULTS, OVERALL CUMULATIVE SUMMARY FOR THE MAIN MODEL
yall[1,] <- coef(crall)
Sall[[1]] <- vcov(crall)

# Inland

{# Cross bases and first stage models defined above
# REDUCTION TO SUMMARY ASSOCIATIONS, TO OVERALL CUMULATIVE SUMMARY
crall <- crossreduce(cb3, Mod_31_, lag = c(0,2))

# STORE THE RESULTS, OVERALL CUMULATIVE SUMMARY FOR THE MAIN MODEL
yall[2,] <- coef(crall)
Sall[[2]] <- vcov(crall) }

method <- "reml"
mvall <- mvmeta(yall~1,Sall,method=method)
summary(mvall)

xvar <- seq(bound[1],bound[2],by=0.1)
bvar <- do.call("onebasis",c(list(x=xvar),attr(cb,"argvar")))
xlag <- 0:100/10
blag <- do.call("onebasis",c(list(x=xlag),attr(cb,"arglag")))

cpall <- crosspred(bvar,coef=coef(mvall),vcov=vcov(mvall),
 model.link="log",by=0.1,from=bound[1],to=bound[2])

plot(cpall, main = "Meta lag 2", ylim = ylim)

#Estimates
RRM2 <- cpall$allRRfit[which(as.numeric(names(cpall$allRRfit))%in%q.m)]
RRM2se <- cpall$allse[which(as.numeric(names(cpall$allse))%in%q.m)]
RRM2est <- c((RRM2[2])/(RRM2[1]),
 exp( ( log(RRM2[2])- log(RRM2[1]) - (qnorm(.975)*(sqrt( (RRM2se[2])^2+(RRM2se[1])^2) ) ) ) ),
 exp( ( log(RRM2[2])- log(RRM2[1]) + (qnorm(.975)*(sqrt( (RRM2se[2])^2+(RRM2se[1])^2) ) ) ) )) }
