# Supplementary material for: High Summer Temperatures and Mortality in Estonia
Source: PLoS One. 2016 May 11;11(5):e0155045. doi: 10.1371/journal.pone.0155045 (PMC4864204; doi:10.1371/journal.pone.0155045)
Supplement: S1 Table — (DOCX) [file pone.0155045.s003.docx]

S1 Table. Descriptive statistics for the daily maximum temperatures for the summer months over the period 1997–2013 per meteorological station

| REGION | STATION | MEAN | SD | MIN | MAX | 75^th^ percentile | 90^th^ percentile | 99^th^ percentile |
| --- | --- | --- | --- | --- | --- | --- | --- | --- |
| COASTAL | PÄRNU | 20.7 | 4.3 | 6.6 | 33.2 | 23.3 | 26.7 | 30.7 |
| COASTAL | TALLIN | 19.8 | 4.2 | 6.9 | 32.3 | 22.5 | 25.5 | 30.0 |
| COASTAL | LAANE-NIGULA | 20.5 | 4.4 | 7.6 | 33.3 | 23.3 | 26.3 | 30.5 |
| INLAND | TORAVERE | 20.7 | 4.6 | 6.2 | 34.2 | 23.7 | 26.8 | 30.8 |
| INLAND | VORU | 21.0 | 4.8 | 5.9 | 35.1 | 24.2 | 27.3 | 31.8 |
| INLAND | JOHVI | 19.9 | 4.6 | 5.3 | 34.6 | 23.0 | 26.1 | 30.6 |
